# Asepsis in Dentistry

**Published:** 1911-04-15

**Authors:** Joseph S. Graham


### ASEPSIS IN DENTISTRY.

BY JOSEPH S. GRAHAM, M.B., M.R.C.S.

In bringing this subject before you I feel that I shall have views to express which may be considered extreme by some of the older men in your profession—by men who have done so much to establish the science of dentistry and to place it where it belongs, among the learned professions.

We must consider two points. Firstly, what are the present methods in daily use: and secondly, whether it is necessary that these methods be changed? Perhaps it would be easier to show you how these present methods impress one not versed in the mysteries of your craft, but say, with a knowledge more or less intimate, of bacteriology. I go to my dentist and find him immaculately dressed in his washable linen coat, fresh shaven, and clean hands and well-trimmed finger nails. I seat myself in the dental chair, not knowing who may have been there before me, but content with any slight risk I may run, as one of the daily chances I am prepared to take. The dentist invites me to put my head on the head-rest, where I find a clean linen doylie. I might sooner have a towel fresh from his cabinet to rest my head on, but again I take greater chances during the course of an ordinary day.

Now we are ready for work. A small mirror is wiped off with a towel or wipe and inserted in my mouth. This patient is looking for what strikes him as faults, or is over-sensitive, and he wonders in what type of mouth that mirror was last. Is it surgically clean? If this can be answered in the affirmative he is happy, for this is not one of the every day chances of life. After the examination the dentist takes some instruments from a dish partially filled with a fluid, which his patient finds to be a formaldehyde solution, usually employed for sterilizing the instruments, as there is less tendency to the formation of rust. The operator may or may not wipe the disinfectant from the instruments, and places them on the flat surface of his swing instrument cabinet. Now the patient wonders whether this surface has been sterilized after the instruments from the last case have been removed. He asks as much, and is told that for finicky people he keeps some squares of blotting paper in his cabinet, and that the blotting paper has the added advantage of absorbing any moisture. This, is, of course, impossible of sterilization, but at any rate is free from the material removed from the mouths of previous patients.

Say our dentist has outlined the work necessary to be done and is now ready to prepare a cavity for a filling. We take it that he chisels away the decayed material instead of immediately using a burr. This decayed and frequently septic material he may not be able to remove from the cavity by his instrument, so that he next takes a small wisp of cotton on a pair of cotton pliers and revolves the pliers so that the dead material in the cavity may adhere to the cotton. Now, in order to remove this fine sponge from the pliers, he uses a little nickel waste receptacle, inserts his pliers into one of the arms cut on the upper surface, and withdrawing the pliers leaves the wipe in the receptacle and incidentally covers the pliers with bacteria from the mouths of previous patients. This, unfortunately, is a very common practice. Having prepared roughly his cavity, it is now time for the use of a burr. The dentist selects from a drawer in his swing cabinet a burr or series of burrs, and proceeds to clean the one he has selected on a revolving wire brush, not with the idea of sterilization but rather that the cutting edges may be cleaned of any detritus. Asked if burrs are sterilized, he answers that the use of an aqueous solution would cause the cogs in the socket to become rusty and a valuable instrument would be of no further service.

So much for the preparation of an ordinary cavity, and, providing none of the instrumenst have slipped during the operation, causing an abrasion of the mucous membrane, no immediate result will follow and the cavity is well filled. However, is it not possible that the dentist has added to this patient's mouth organisms from the mouths of other individuals? Organisms possibly of marked virulence, organisms proficient in the art of splitting up starches, pyorrhea organisms, etc., which may find a suitable medium for growth amid new surroundings.

This is a source of danger from these types of bacteria, but the possibility of the introduction of syphilis, tuberculosis, scarlet fever, typhoid fever, etc., is, of course, always present and a danger which may not be overlooked. Even though only one patient out of a thousand be saved from any of these diseases, a more elaborate technique is justifiable. Do any patients suffering from syphilis seek the dentists aid? Assuredly they do. One of the first things a good physician does before starting his patient on a course of mercury is to see that the teeth are in good condition and it is an even chance that he does not inform the dentist of the condition of affairs. Is he a good physician in this respect? Certainly not; and unfortunately the dentist does not often see a mucous patch which may be hard to recognize without the history of the patient and the knowledge of a primary sore. This knowledge the patient is going to keep to himself.

A word or two about tuberculosis. The probability of the introduction of tubercle bacilli from the mouth of a tuberculosis patient to that of a healthy individual may be slight, but possible. The danger to the dentist who, leading an indoor life and working with his face close to that of the patient, is great, both from the breath and the spray which results from coughing by the patient, and from the fact that his room has become infected. We will take up later what seems to be a simple method by which this danger may be minimized.

Let us now take up the question of extraction. Practically all dentists boil their forceps, mouth gag, etc., but do they always prepare the field of operation? We know of some people who were referred by their own dentist to an extraction specialist, without any advice as to the preparation of the mouth. After extraction advice is almost invariably given, with a prescription for some antiseptic mouth wash, both of which are doubtless of benefit, as we see but few cases "go bad" after this operation. But surely the sane and safe way is the best, and if some of these few cases can be spared an infection it is again worth while.

In going over this we have only touched on the possibility of the spread of infection from one mouth to another, and have said nothing of the defensive agents of the body, which, fortunately for us, are great. I fancy I can see some busy practitioners smile and remark that times have changed—that they have never seen any bad effect due to their treatment (or anything at least that could be attributed to them)—but these men can not deny the possibility of transference of infection, which we see in the other fields of medicine. Now we may ask ourselves whether it is necessary that other methods be adapted. My answer would be that it is. If our cutting instruments are not perfectly sterilized, or if perfectly sterile, laid on an unsterilized surface, we may transfer infection to the mouth surface. If one of these instruments should slip and an abrasion be made we may have serious results, for we know that in cavity formation we are dealing with organisms which ordinarily require oxygen for their growth, but when growing in the deeper parts of a cavity have practically become anerobic, and sometimes of great virulence. If these organisms gain entrance to the body below the mucous membrane we may have serious results. You may ask why it is necessary to be so careful beforehand when you are dealing with septic cavities and the sterile instruments will so soon become septic. You may argue that if an instrument slip and cause an abrasion it is likely to do so after being used in a septic cavity, and that even if the cavity were sterile, organisms might be carried from the surface of the mouth deep into the tissues below the epithelium, which, of course, is one of the chief defences of the body.

My argument would be this: 1st. That we may transfer through non-sterile instruments diseases such as typhoid fever, tuberculosis, syphilis, etc. 2nd. That we may introduce organisms of the caries-producing type, and these may be of great virulence; and 3rd. In the case of abrasions, there is no need to increase the risk by having our instruments septic beforehand, as it is impossible to gauge immediately the virulence of the organisms on our instruments, or to estimate the immunity of our patients to organisms brought from an outside source.

The ideal aseptic conditions of course would be such as are carried out in the operating theatre of a hospital. The operating room is as aseptic as it is possible to be made by disinfection. The patient has been thoroughly prepared, the parts to be operated on are aseptic, the surgeon in gown, with head, mouth and nose covered, and wearing sterile gloves. Finally, sterile instruments and towels. Such a condition of affairs, while to be desired, is quite unnecessary and impracticable. What we must aim to do is to prevent transference of bacteria from one mouth to another, to protect the patient from the dangers of infection should we wound the tissues either intentionally or unintentionally, and to protect ourselves from infection by the patient. Let me impress upon you once more that such procedures as we shall outline are not difficult to carry out, are not "faddy," and that once you get into the habit of taking these precautions for your own and your patient's sake they will come as natural to you as does personal cleanliness. Surgical cleanliness is just one step further. Let us consider then the various points and how this routine may easily be carried out.

### THE OPERATING ROOM.

Sunlight and ventilation must be two factors to be taken into consideration in the choice of an office. Sunlight is the best natural germicide we have, as, while the majority of bacteria are inhibited in growth by the action of bright daylight, all are inhibited by direct sunlight, and if this action be prolonged lose the power of developing when placed in the dark. We must thus choose an office with the maximum of sunlight, if only for our own protection, and it is better for us living in the northern part of the world to have an office with windows facing both east and west, as giving the maximum of sunlight during the winter time, when our ventilation must of necessity be poorer than in the summer. I do not mean by this that our windows should be shut in the winter time. A piece of board eight inches in height placed across the lower part of the window sash and on the inside of the room allows of the window being opened two or three inches from the bottom without the danger of a draught. The window on the other side of the room may be slightly open at the top. We assure ourselves of health by this means, and especially is this of importance to the dentist, he doing so much indoor work and with so little chance of outdoor recreation. There may be objections raised to the heat of such a room in the summer time, but with inside shutters on the windows, this objection may be dispensed with.

The office finish should be as simple as possible. Hardwood floors (preferably with rounded corners where the wall meets the floor) are a necessity, or, if this be impossible, a linoleum covering for the floor is to be preferred to carpet. Hardwood or linoleum can easily be washed and kept clean, and if we have the rounded corners there is no possibility of dust collection. The walls and ceiling should be covered with a sanitary paper, so called on account of the ease with which this may be washed. The whole object is to have things as simple as possible, and a finish which may easily be disinfected at regular intervals.

### FIXTURES.

The complicated dental chair leaves much to be desired as regards simplicity. This, however, I think we will have to accept as a wonderful work of art. The swing cabinet allows of change in a good many instances. It should always have a glass top which can be sterilized before sterile instruments are laid on it. For those who already have a wood swing table this may be accomplished by the purchase of a square of plate glass cut to fit the upper surface of the table. If there are drawers in the swing cabinet, they should be lined with porcelain or a hard, white enamel, in order that they may be readily cleaned by moist dusting. The same may be said of the drawers of the instrument cabinet. The idea of course is to have everything so constructed as to be easily washed and with as few receptacles as possible for the collection of dust.

### OFFICE DISINFECTION.

It is a well known fact that in laboratories where experimental work is done on animals, the operating rooms become septic so that wounds do not heal by first intention, making it necessary to abandon the room for some time. In all larger hospitals this fact is reckoned with, and an operating room is set aside for "dirty" cases. Now room for infection is most likely to occur in a dental office where work is being carried on in septic mouths. It has been well said that, surgically speaking, the mouth is the dirtiest cavity in the body. This being granted, it is necessary that a dental office should have systematic disinfection, for the protection of the patients, as well as for the protection of the operator and his family. Added to the risk of sepsis following operations necessitating the wounding of mucous membranes, there is the risk of infectious diseases such as scarlet fever, diphtheria, measles, etc. These dangers may, to a great extent, be avoided by disinfection. A practical way is to have a thorough disinfection once a month. Take say the first week end. Seal on Saturday night the doors and windows with strips of paper covering the cracks. This may be done by using a starch paste that can easily be washed off. Open the drawers containing the instruments, and have all fires extinguished in the room, not that there is any danger of explosion, but because the room has to remain closed for twenty-four hours. Place in the centre of the room a jar containing 10 oz. of formaldehyde, add to this, 6 oz. of potassium permanganate, and beat a hasty retreat, for the gas is generated exceedingly rapidly. The door of egress should then be sealed and the room allowed to stay closed until Monday morning, when the windows may be opened and the room aired. This proceeding is not hard to carry out, and the extra work entailed will not be noticed if you make it a habit. For wet dusting 1-1000 bichloride solution may be used; this includes the walls and the floors. If this latter were done weekly it should be sufficient.

PREPARATION FOR DENTAL OPERATIONS.

Instruments should be sterilized before all operations, whether wounding of the mucous membrane is intended or not. This is best done by boiling. It is very simple where there is electricity in the office, to have a sterilizer heated by electricity, or to have an ordinary sterilizer, six by four, heated by a Bunsen burner or an alcohol lamp. It is well to allow the instruments to boil for five or ten minutes, remove by means of a false bottom, then place them (if not wanted immediately) in a weak formalin solution and dry before using with a sterilized cotton wipe. Many dentists use a formalin solution and do not boil, but experiments in this show that instruments infected with a vigorous growth of staphylococcus, if placed in a tight box and exposed for 15 hours to the vapors of formaldehyde, generated by evaporation from a large shallow dish of formaldehyde (40%) and then washed in sterilized water, showed a good growth in broth. A formalin solution is a good disinfectant in which to place sterilized instruments, and if expense is not a prime factor, I would suggest that two sets of cutting instruments be employed so that there would be no delay between appointments. Burrs or cutting instruments used in the dental engine may be sterilized by boiling in oil, then transferred to Benzol or wiped off by a sterilized wipe dipped in benzol. There is no danger of water getting into the hand piece and ruining it, and no danger from the wire brush; this latter, if a necessity, may be sterilized by boiling in oil or water, then dipped in benzol or gasoline to remove the oil or water. The benzol may be kept in a flat dish with a ground glass cover, say with a diameter of four inches. Burrs may be left in this solution without danger of rust. The dish containing the benzol or gasoline should not be opened beside a flame. The instruments intended for use on a patient should be dried by means of a sterilized wipe, or in case of burrs by dipping in benzol or gasoline which evaporates quickly. The instruments may now be placed on the glass surface of the swing table which has already been sterilized by wiping with 1-1000 bichloride, or on a sterile towel placed on the surface of the table. I would recommend boiling instruments rather than any other method of disinfection, as it is much easier to do and has the further advantage of being non toxic. A series of experiments, made in the Laboratory of the Board of Health, 1903, showed that infected instruments placed in pure carbolic (95%) for 10, 15, 30, 60, 90 seconds, and then washed in sterilized water (not wiped) and afterwards placed in a broth tube, showed a growth from instruments immersed for 10, 15, 30 and 60 seconds.

In case a smooth cutting instrument be needed for immediate use, and not previously sterilized by boiling, a pretty sure method is to wipe off with pressure by means of sterilized wipe dipped in alcohol.

### ABSORBENT COTTON WIPES, TOWELS, DRESSING, ETC.

It is just as convenient to use sterilized absorbent as non-sterile. Wipes may be made of absorbent cotton gathered together in little balls, about one inch in diameter, and wrapped in plain factory cotton, perhaps 10 or 15 wipes in a bundle, pinned and placed in the oven of the kitchen range for sterilization. The same may be done with towels, but in this case I would recommend the use of cheap towels as scorching may take place. You may have a steam sterilizer placed in your office at a very moderate cost ($25.00 or $30.00), which would do away with the possibility of scorching, as it is perfectly safe to sterilize the finest linen by this method. The advantages are that you have a sterile towel to place under the head of your patient a sterile towel for his neck and sterile cotton to place on sterile instruments.

### THE OPERATOR.

Little need to be said as to personal cleanliness and the use of a washable linen coat. These are matters of habit on the part of the dental operator. The nails should be kept short, and care should be taken to have no hang nails. The hands should be well scrubbed in running water for a couple of minutes before work on each patient is begun. The use of rubber gloves is unnecessary, except in cutting operations. There is one matter of great importance, and that is the covering of the mouth and nose of the dentist by three or four thicknesses of gauze, which may be tied at the back of the head. This allows of the air breathed by the dentist to be filtered by the gauze. The physician, in examining the chest of a patient, requests him to turn away the head, so that he need not breathe the directly expired air of the patient, but the dentist, working as he does, must necessarily breathe a certain amount of the expired air. This is not all. A patient in the dental chair may find it impossible to restrain a cough, and a fine spray is emitted, which strikes the dentists' face. In the case of tuberculosis, the expired air is not nearly of such great danger as the spray which results from coughing, when many tubercle bacilli are set free in the air. If placing of gauze over the mouth and nose is not made a routine practice, it is strongly recommended in doubtful cases.

### THE MOUTH.

In septic mouths, or in mouths where a cutting operation is to be done, a preparation of the field of operation is necessary. There is no safer mouth wash that I know of than potassium permanganate solution, used frequently before the appointment. A solution of 1-4000 freely used will do much to render infection less liable to occur. Unintentional cuts through the mucous membrane of the patient, or through the skin of the operator may safely be cauterized by pure carbolic and neutralized in a second or two by the application of alcohol to the cauterized area. Too much care cannot be taken in this regard.

### RECEPTACLES FOR WASTE.

Mention has been made before of the nickel repository for waste. This is bad, as we do away with many of the benefits of sterilization. All receptacles should be made of cardboard or paper and burnt after each patient.

### CARE OF INSTRUMENTS.

After use, instruments should be boiled, wiped dry and put away in drawers especially prepared for them. It has been said that boiling dulls the edges of cutting instruments, but it is hardly possible to conceive how this should be the case unless it were due to rubbing against other instruments. This may be avoided by rolling a wisp of cotton around the cutting edge before sterilizing.

It takes a little extra time and a little extra care on the part of the dentist to carry out such a technique, and there comes the practical question as to who is going to pay. The patient of course, and gladly, for the public are fast becoming educated along the lines of prevention. Take as an example the question of certified milk with the extra expense involved in its production. Many of us pay willingly for the extra security. Help in the education of the public. If sometimes it is unappreciated by them, you will at least have that glow of satisfaction that comes from duty well performed.—*Oral Health.*

